# Bone biopsies guided by augmented reality: a pilot study

**DOI:** 10.1186/s41747-023-00353-w

**Published:** 2023-07-20

**Authors:** Domenico Albano, Carmelo Messina, Salvatore Gitto, Vito Chianca, Luca Maria Sconfienza

**Affiliations:** 1 IRCCS Istituto Ortopedico Galeazzi, Milan, 20161 Italy; 2 Dipartimento di Scienze Biomediche per la Salute, Università degli Studi di Milano, Milan, 20122 Italy; 3 Clinica Di Radiologia EOC IIMSI, Lugano, Switzerland; 4 Ospedale Evangelico Betania, Via Argine 604, Naples, 80147 Italy

**Keywords:** Augmented reality, Bone, Image-guided biopsy, Radiology (interventional), Tomography (x-ray computed)

## Abstract

**Purpose:**

To test the technical feasibility of an augmented reality (AR) navigation system to guide bone biopsies.

**Methods:**

We enrolled patients subjected to percutaneous computed tomography (CT)-guided bone biopsy using a novel AR navigation system. Data from prospectively enrolled patients (AR group) were compared with data obtained retrospectively from previous standard CT-guided bone biopsies (control group). We evaluated the following: procedure duration, number of CT passes, patient’s radiation dose (dose-length product), complications, and specimen adequacy. Technical success was defined as the ability to complete the procedure as planned, reaching the target center. Technical efficacy was assessed evaluating specimen adequacy.

**Results:**

Eight patients (4 males) aged 58 ± 24 years (mean ± standard deviation) were enrolled in the AR group and compared with 8 controls (4 males) aged 60 ± 15 years. No complications were observed. Procedure duration, number of CT passes, and radiation dose were 22 ± 5 min, 4 (median) [4, 6 interquartile range] and 1,034 ± 672 mGy*cm for the AR group and 23 ± 5 min, 9 [7.75, 11.25], and 1,954 ± 993 mGy*cm for controls, respectively. No significant differences were observed for procedure duration (*p* = 0.878). Conversely, number of CT passes and radiation doses were significantly lower for the AR group (*p* < 0.001 and *p* = 0.021, respectively). Technical success and technical efficacy were 100% for both groups.

**Conclusions:**

This AR navigation system is safe, feasible, and effective; it can decrease radiation exposure and number of CT passes during bone biopsies without increasing duration time.

**Relevance statement:**

This augmented reality (AR) navigation system is a safe and feasible guidance for bone biopsies; it may ensure a decrease in the number of CT passes and patient’s radiation dose.

**Key points:**

• This AR navigation system is a safe guidance for bone biopsies.

• It ensures decrease of number of CT passes and patient’s radiation exposure.

• Procedure duration was similar to that of standard CT-guided biopsy.

• Technical success was 100% as in all patients the target was reached.

• Technical efficacy was 100% as the specimen was adequate in all patients.

**Graphical Abstract:**

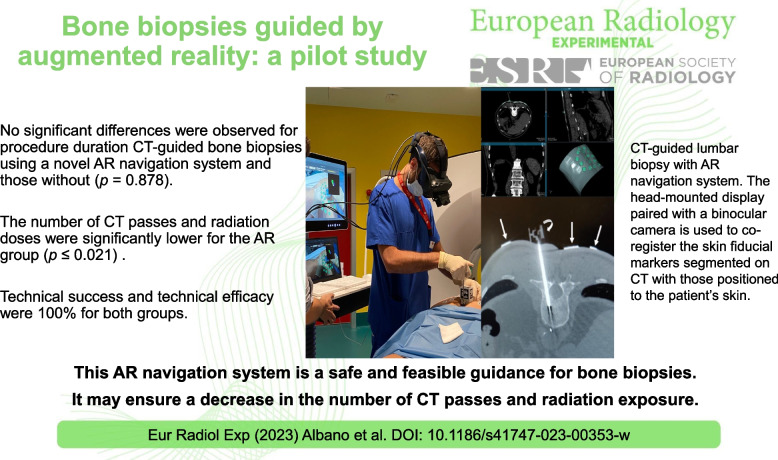

## Background

Imaging is essential in the diagnostic work-up of bone disorders, being also necessary as a guidance for biopsies of focal and diffuse bone lesions to obtain the final diagnosis [1–3]. Computed tomography (CT) is the best guidance when the cortical bone is intact or the lesion is particularly deep. It allows monitoring the needle path to reach the target lesion, avoiding nearby structures [4]. Nevertheless, CT guidance has non-negligible limitations: (i) the needle trajectory cannot be visualized real time unless CT fluoroscopy is used, which exposes both patient and operator to high radiation dose, (ii) its low contrast resolution in the soft tissues limits the visualization of vessels and nerves, and (iii) radiation exposure may be substantial, mostly being dependent on the number of scans performed during the procedure. Magnetic resonance imaging (MRI) has the advantages of avoiding radiation exposure also presenting higher contrast resolution in the soft tissues, but high costs, long procedural times, and the need for specific equipment have limited its application as a guidance for bone interventions [5]. Even ultrasound avoids radiation exposure and ensures real-time monitoring of needle trajectory [6–9] but cannot be used when the bone cortex is intact [10].

Several navigational system tools have been introduced to improve the accuracy, reliability, and safety of conventional imaging guidance [11, 12]. Image fusion platforms are based on electromagnetic or optical devices [13, 14], CT with laser marker systems [15], CT fluoroscopy [16], cone-beam CT [17], CT with electromagnetic tracking [18], and robotic systems [19] have been introduced in routine daily activity of several institutions. Nevertheless, these systems still present non-negligible drawbacks, including the inability to ensure a real-time (three-dimensional) 3D visualization of the needle, target, and nearby structures, together with the need to move the operator’s gaze from the interventional field and the screen [20]. Recently, technology advancements have led to the development of augmented reality (AR) navigation systems, which allows for real-time interaction by the operator, overlaying digital content onto the visualized real setting through a specific optical device [21, 22]. AR has been used to augment anatomical and pathological structures by creating 3D anatomic volumes from cross-sectional images and overlapping them over patients using advanced tracking systems (like electromagnetic or optical). Previous studies have tested AR on phantoms to guide percutaneous interventions [23, 24], but recently, this technology has been shown to be accurate and safe for guiding percutaneous thermal ablation of liver tumors in humans [25]. However, none of these studies has specifically dealt with bone biopsies.

Routinely, percutaneous CT-guided biopsies are performed as standard procedures for guideline-based diagnosis and subsequent therapy in the care of patients with bone tumors. Nevertheless, some drawback of these interventions should be pointed out. CT scans must be done repeatedly during a CT-guided percutaneous biopsy, to monitor the advancement of the needle, with a non-negligible amount of ionizing radiations. Furthermore, needle tracking and real-time image acquisition cannot be done, so that CT guided requires imaging interruption every time the patient is moved away from the gantry. AR navigation systems to guide bone biopsies have the potential of decreasing the number of CT passes to track the needle (thereby reducing radiation exposure) and procedure duration and also increasing the safety and efficacy of the procedure. Since a feasibility study on this relatively novel navigation system is needed before proceeding with a prospective randomized controlled study, the aim of this pilot study was to test the technical feasibility of Endosight, an advanced AR navigation system, in guiding a biopsy needle to bone lesions of the axial skeletal system.

## Methods

### Study design

This single-center cross-sectional prospective study was approved by our ethical committee (Protocol: ESBB1, Code number: NCT05732558, approved in 12 October 2022 by Ospedale San Raffaele, Milano, Italy), and all participants provided written informed consent for collection of data to be used for scientific purposes. After matching imaging and clinical data, our database was anonymized to remove any connections between data and patients’ identity according to the General Data Protection Regulation for Research Hospitals.

We prospectively enrolled consecutive patients admitted to the Radiology Department of IRCCS Ospedale Galeazzi-Sant’Ambrogio, Milan, Italy, to be subjected to percutaneous CT-guided bone biopsy in December 2022 and January 2023. Included patients were affected by bone lesion of the axial skeletal system and provided written informed consent to participate in the study. Exclusions criteria were as follows: (i) age < 18 years, (ii) pregnancy, (iii) breast feeding, and (iv) coagulation impairment. Data from prospectively enrolled patients (AR group) were compared with data obtained retrospectively from previous CT-guided bone biopsies performed at our institution between September 2021 and July 2022 (control group). In August 2022, our institution moved to another hospital. After that, there was a long period in which, due to internal rearrangement of our activities, CT-guided biopsies were performed only by orthopedic surgeons up to the start of this study. So, we preferred to include in the control group the last CT-guided biopsies done by the same radiologist that performed the biopsies of the AR group. To do that, our institutional database was searched for lesions with similar features of those included in AR group, *i.e.*, patient of similar age and sex, similar anatomical location, similar size, and similar appearance (sclerotic/lytic, intact/disrupted cortical bone), subjected to conventional CT-guided biopsy. Patients from control group were considered as controls in this study.

### Biopsy guided by the AR system

**Fig. 1:**
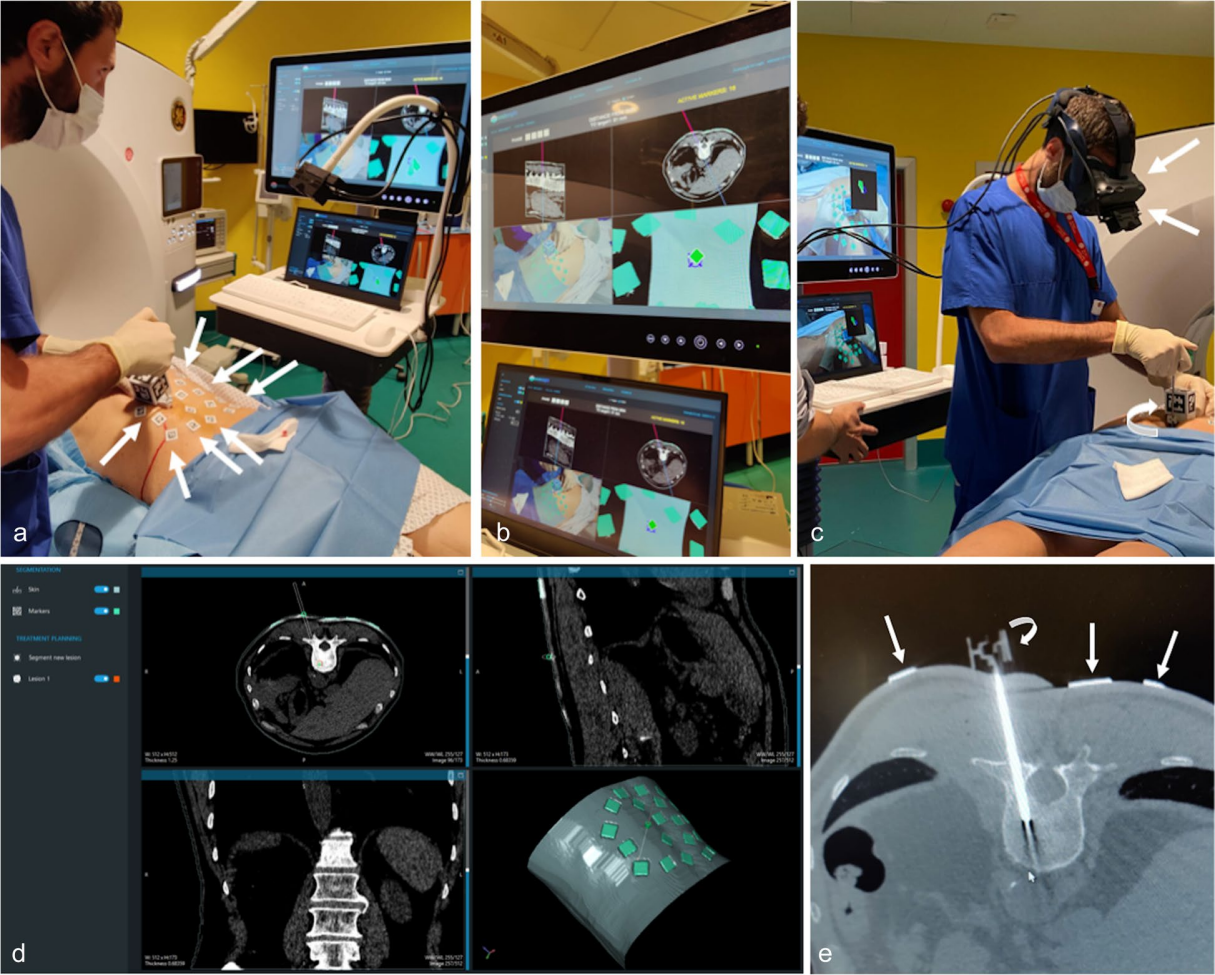
A representative case with lumbar bone lesion from our study population. The fiducial markers are placed on patient’s skin around the entry point of percutaneous biopsy (arrows in **a** and **e**). The AR system (Endosight, R.A.W. Srl, Milan, Italy) consists of a 27″ medical display (ACL, Leipzig, Germany), a laptop (Dell Technologies, Round Rock, TX, USA) with AR software (**b**), and a commercially available head-mounted display (HMD) (Oculus Rift-S, Facebook Technologies, Menlo Park, CA, USA) paired with a binocular camera (Zed Mini, Stereolabs, San Francisco, CA, USA) (arrows in **c**) that is used to coregister the fiducial markers segmented on CT images with those positioned to the patient’s skin (**b**). The best needle trajectory to reach the target is built (**d**). A specific marker attached to the needle to monitor its position and angle during the procedure (curved arrows in **c** and **e**)

All biopsies of AR group were performed in the same CT suite (Revolution Ascend, GE Medical, Milwaukee, MI, USA) by a radiologist with 7 years of experience in CT-guided bone biopsies. The patients were placed on the CT bed based on the location of the bone lesion. After skin disinfection, the first CT scan was acquired after placing 20 specific fiducial Endosight markers on patient’s skin around the entry point of percutaneous biopsy (Fig. 1a and e, arrows). Then, preprocedural CT images were acquired ensuring to include all 20 Endosight markers and uploaded in the Endosight software. The Endosight AR navigation system (Endosight, R.A.W. Srl, Milan, Italy) is a CE marked system composed by a 27″ medical liquid crystal display (ACL, Leipzig, Germany), a high-performance laptop (Schenker DTR17 Schenker Technologies GmbH, Leipzig, Germany) equipped with Endosight software (Fig. 1b), and a commercially available head-mounted display (HMD) (HTC Vive Pro 2, HTC Corporation, Taoyuan C, Taiwan) paired with a binocular camera Basler Dart, Basler AG, Ahrensburg, Germany) (Fig. 1c, arrows). After uploading the pre-operation CT images in Endosight software, the volume of all anatomical structures is created allowing to recognize and highlight the target lesion, and to plan the needle path, together with its length and diameter (Fig. 1d). Then, the portion of patient’s skin is captured by the binocular cameras allowing the software to co-register all the fiducial Endosight markers applied to the patient’s skin with those previously segmented on the CT images (Fig. 1b). Endosight software is designed for two modes, liver and quick intervention. While liver mode is specifically designed for ablation procedures on the liver, the quick-intervention mode can be adapted to any percutaneous procedures involving a guided needle insertion. In the quick-intervention mode, used within this study, the software provided 3D reconstruction and automatic segmentation (from CT scans to 3D volumes) of the skin and of Endosight markers, and a semiautomatic segmentation of the target lesions. To monitor the correct position and angle of the needle during the procedure, an Endosight sensor was attached, thanks to a dedicated clamp, to the 8-G needle (15 cm in length, Jamshidi 8 G, HS Hospital Service, Italy), which was used to biopsy the bone lesion (Fig. 1c and e, curved arrow). The same radiologist performed all standard CT-guided bone biopsies of controls in a different CT suite, a 64-section CT system (Somatom Emotion, Siemens Medical Solutions, Erlangen, Germany).

### Data collection, sample size calculation, and statistical analysis

For each biopsy, we collected the following data:

- elapsed time from local anesthesia to specimen withdrawal;
- number of CT passes;
- radiation dose administered to the patient measured by dose-length product (DLP, mGy*cm);
- complications;
- specimen adequacy;
- height, weight, and body mass index (BMI) of AR group patients.

Concerning the retrospective evaluation of procedure duration, as per our routine during CT-guided bone biopsies, we use to acquire a first CT scan to show the correct position of the needle inserted for injecting local anesthesia and a last CT scan to show the final position of the needle that is used to biopsy the bone lesion. Hence, we can easily calculate the elapsed time from local anesthesia to specimen withdrawal just looking at the time of acquisition of the first and last CT images. Furthermore, we use to report the time of local anesthesia and the time of end of the procedure in a specific form.

Technical success was defined as the ability to complete the procedure as initially planned [11, 26] to reach the target lesion and to obtain a bioptic sample. Technical efficacy was assessed on the basis of specimen adequacy, as determined by the pathology report. Differences in terms of demographics, lesion characteristics, and procedure details were investigated between AR group and control group.

In order to compute the sample size for this pilot study, the following formula was used [27]:

$$n= \frac{{\sigma }_{1}^{2}{({z}_{1-\frac{\alpha }{2}}+{z}_{1-\beta })}^{2}m}{m{\Delta }^{2}-{({z}_{1-\frac{\alpha }{2}}+{z}_{1-\beta })}^{2}{\sigma }_{0}^{2}} .$$

Parameters *Δ* and ${\sigma }_{\mathrm{0,1}}$ represent, respectively, the difference between the means of the two groups and their standard deviation. This study targets a minimum difference of *Δ* = 3 scans, with ${\sigma }_{0}$ = ${\sigma }_{1}$  = 2. A significance level of *α* = 0.05 and a power of the test of 1-β = 0.8 are considered. The number m of patients belonging to the historical control group is set as *m* = 7 patients. The formula gives a number *n* of prospective patients to be included in the study as *n* = 7. Considering a possible dropout rate of 10%, the final total number of patients included in the study is found to be 16, with *n* = 8 and *m* = 8.

Anonymous data were analyzed using MATLAB 2016b (the *MathWorks*, Natick, MA, USA). Differences among variables were evaluated by Fisher’s exact test for categorical variables, and by nonparametric Mann-Whitney *U*-test for continuous variables. A *p*-value < 0.05 was considered to indicate statistical significance. Categorical variables were reported as absolute value and percentage; continuous variables were reported as either mean ± standard deviation or median and interquartile (25th–75th) range.

## Results

According to our criteria, 8 patients (4 males, 4 females; aged 58 ± 24 years; range 19–83) were prospectively enrolled in AR group, and 8 patients (4 males, 4 females; aged 60 ± 15 years; range 35−74) were retrospectively included in control group. The mean height, weight, and BMI were 173 ± 9 cm, 74 ± 8 kg, and 25 ± 1, respectively. No significant differences in age (*p* = 0.959), gender (*p* > 0.999), or lesion characteristics (*p* > 0.999) were observed among the patients and controls. No complications were observed in both groups. Procedure duration, number of CT passes, and radiation dose were 22 ± 5 min, 4 (4, 6), and 1,034 ± 672 mGy*cm for the AR group and 23 ± 5 min, 9 [7.75, 11.25], and 1,954 ± 993 mGy*cm for the control group, respectively. No significant differences between the two groups were observed in terms of procedure duration (*p* = 0.878). Conversely, number of CT passes and radiation dose were significantly lower for the AR group than for controls (*p* < 0.001 and *p* = 0.021, respectively). Patient demographics, lesion characteristics, and procedure details are reported in Table 1.

**Table 1:** Full data of all patients (AR group) and controls (control group)

**Gender**	**Age (y)**	**Location**	**CT passes**	Dose (mGy*cm)	**Duration (min)**	**Pathology report**
**AR group**						
F	52	Pelvis	4	605.43	33	Negative for cancer
F	56	L3	4	946.00	19	Spondylodiscitis
M	80	Pelvis	6	773.43	22	Metastasis
M	67	L1	6	326.35	21	Metastasis
F	75	Scapula	4	775.00	22	Multiple myeloma
F	83	Pelvis	4	1427.50	23	Metastasis
M	19	Pelvis	4	912.46	17	Chordoma
M	28	Pelvis	7	2504.00	21	Hemangioma
**Control group**						
M	61	Pelvis	8	1927.00	18	Metastasis
F	39	L1	8	1089.00	20	Hemangioma
F	74	Pelvis	7	1531.00	20	Osteoblastoma
M	35	L2	7	919.00	17	Spondylodiscitis
F	63	Scapula	11	2287.00	25	Chondrosarcoma
M	64	Pelvis	12	2068.00	27	Multiple myeloma
F	69	Pelvis	10	1686.00	26	Metastasis
M	74	Pelvis	14	4125.00	32	ARMD

*M* Male, *F* Female, *y* Years, *Dose* Radiation dose in DLP, *Duration* Procedure duration time in minutes, *ARMD* Adverse reaction to metal debris in a patient with total hip arthroplasty

Technical success was 100% in both groups, as in all patients the center of the target lesion was reached and the sample was obtained. Technical efficacy was 100% in both groups, as the specimen was adequate in all patients according to the pathology report. Pathological analysis reported: one osteoblastoma, one chordoma, one chondrosarcoma, two myelomas, five metastases (from breast, colon, prostate, lung, and ureteral carcinoma), two hemangiomas, two spondylodiscitis, one negative for cancer, one adverse reaction to metal debris in a patient with total hip arthroplasty.

## Discussion

The main finding of this pilot study was that this novel AR navigation system can be used as a feasible and safe guidance for bone biopsies in the axial skeletal system presenting 100% technical success and efficacy. AR allowed for a significant reduction of radiation dose and number of CT passes required to monitor the needle trajectory, without significant changes in procedural duration time, when compared with standard percutaneous CT-guided biopsy.

Imaging guidance is essential for diagnostic and therapeutic musculoskeletal interventions to improve reliability and safety of the procedure itself [28–30], but CT cannot provide a real-time monitoring of needle advancement without the need of interrupting the procedure to check the needle position. On the other hand, the Endosight AR navigation system, by overlapping 3D anatomic volumes over patients in the real interventional field, ensures a sort of real-time monitoring of needle trajectory, thereby decreasing the need of acquiring several CT passes generally performed to check needle trajectory and to reach the target safely avoiding neurovascular structures. This makes the method safe, as proven by the absence of complications seen in our study. The most important results of this pilot study, in addition to the safety of this AR system, are the feasibility of this tool demonstrated by the complete technical success and technical feasibility observed in all procedures. Indeed, all biopsies performed in the AR group were completed as initially planned to obtain a bioptic sample, and all specimens were adequate, as reported by the pathologist. It means that this navigation system might be used in future randomized studies to be compared with the standard CT-guided percutaneous procedure.

As a matter of fact, the most crucial point for the application of an AR navigation system is the reliability of superimposition of images that can be achieved only with a perfect coregistration. Previous studies have investigated the registration accuracy on phantoms and humans reporting interesting results. Hecht et al. [31] used an AR navigation system to guide needle advancement on phantoms reporting about 2.7-mm mean error of the needle positioning, which resulted 78% lower than the standard CT-guided intervention. Similar improvements were reached also by Long et al. [32] in an abdominal phantom using cone-beam CT-guided fluoroscopy and AR navigation systems. Notably, both studies also reported a substantial decrease of procedure duration.

The need of reliable navigation systems for bone procedures has been already highlighted by other studies recently published, involving spine surgical interventions [33] and CT-guided bone procedures [34]. Solbiati et al. [25] used the same Endosight AR system employed in our study to guide thermal ablation of liver tumors. This system consists of skin fiducial markers attached to the patient before the first CT acquisition, a specific Endosight sensor attached to the needle and a software that allows for segmentation of markers, organs, and target lesion, to coregister virtual and real images to build the best trajectory of the needle that can be followed through a display, HMD, or screen. The authors reported very high accuracy of the antenna tip positioning, with technical success of the ablation obtained in all patients. Furthermore, the procedure time was similar to that of conventional CT-guided interventions performed by expert radiologists.

This AR navigation system has never been tested for bone procedures. In our study, we have shown how it might be applied to decrease patient’s radiation exposure and number of CT passes. The procedure duration time was not different from standard percutaneous CT-guided bone biopsies. These results were achieved despite the time required to place all skin fiducial markers and the need to acquire an additional preprocedural CT scan with large field of view to include all the skin markers. Such preparatory procedures will arguably be streamlined with already planned Endosight upgrades, such as the switch from optical to infrared tracking, which should make the whole setup easier and more straightforward. In spite of this limitation, the procedure time remained low, as well as patient’s radiation dose and number of CT passes were significantly decreased, suggesting that, with some moderate adaptation of the technology to the novel scope of use, the timeframe could be further reduced.

The phenomenon of motion sickness (signs and symptoms related to AR experience using the HMD) is still a potential drawback of immersive AR systems due to possible discrepancies between the visual and vestibular senses [35]. However, it was just a mild issue for the radiologist during the first procedures. Indeed, motion sickness has been recently limited by commercially available HMDs. The patient’s respiratory movement and motion are still one of the most important technical issues, with the risk of shifting of the target relative to the expected site. In our study, we decided to focus on bone lesions located in the axial skeleton reducing the risk of shifting related to the motion of patient’s limbs. Further studies should investigate technical feasibility of this AR system to guide bone biopsies in the appendicular skeleton.

Another potential drawback of this AR system is needle bending due to corrections to the trajectory done during needle advancement within the bone. This might create an iatrogenic shift of few millimeters from the intended trajectory. Furthermore, the Endosight sensor attached to the needle is still quite large and may hamper the advancement of the needle itself in deep lesions in addition to potentially hide the view of some skin markers by the HMD, but this technical issue will be solved soon.

Some other limitations of this system and of our study need to be pointed out. The main limitation of our pilot study was the relatively small sample size, although it was enough according to sample size calculation allowing us to reach statistically significant results. Furthermore, this study is valid only as a pilot study indicating safety and efficacy and not as a controlled study. Notably, the CT protocol for guiding percutaneous bone biopsies installed in the two CT units was the same, so the required radiation dose can be just slightly affected by the different machine, while the different device manufacturers cannot impact on the required time of the whole procedure, which is totally dependent on the work of the radiologist. Last, given that it was not a prospective randomized controlled study, we tried to select similar biopsies to be included in the control group based on age, sex, lesion location, size, and appearance (lytic/sclerotic, intact/disrupted cortical bone), precisely to reduce the bias related to different conditions due to patients’ characteristics. We acknowledge that it might be considered a selection bias, but it was the only way to try to make homogeneous the two groups of patients.

In conclusion, this AR navigation system is a safe and feasible tool that can be helpful to decrease patient’s radiation dose and number of CT passes during bone biopsies without increasing the procedural duration time. Although further technological advancements could improve the adoption in the clinical practice, these results encourage further investigation of applications in the musculoskeletal system. Future prospective randomized controlled trials will aim to better understand the real advantages and disadvantages of AR-guided bone biopsies over standard percutaneous CT-guided procedures.

## Data Availability

The datasets used and analyzed during the current study are available from the corresponding author on reasonable request.

## Abbreviations

3D: Three-dimensional
AR: Augmented reality
BMI: Body mass index
CT: Computed tomography
HMD: Head-mounted display
